# Monitoring and evaluating the implementation of essential packages of health services

**DOI:** 10.1136/bmjgh-2022-010726

**Published:** 2023-03-28

**Authors:** Kristen Danforth, Ahsan Maqbool Ahmad, Karl Blanchet, Muhammad Khalid, Arianna Rubin Means, Solomon Tessema Memirie, Ala Alwan, David Watkins

**Affiliations:** 1 Department of Global Health, University of Washington, Seattle, Washington, USA; 2 Center for Global Public Health, Islamabad, Pakistan; 3 Department of Community Health Sciences, Institute for Global Public Health, University of Manitoba, Winnipeg, Manitoba, Canada; 4 Faculty of Medicine, Geneva Centre of Humanitarian Studies, University of Geneva, Geneve, Switzerland; 5 Health Planning Systems Strengthening and Information Analysis Unit (HPSIU), Ministry of National Health Services Regulations and Coordination, Islamabad, Pakistan; 6 College of Health Sciences, Addis Center for Ethics and Priority Setting, Addis Ababa University, Addis Ababa, Ethiopia; 7 DCP3 Country Translation Project, London School of Hygiene & Tropical Medicine, London, UK; 8 Division of General Internal Medicine, Department of Medicine, University of Washington, Seattle, Washington, USA

**Keywords:** health policy, health systems evaluation

## Abstract

Essential packages of health services (EPHS) are a critical tool for achieving universal health coverage, especially in low-income and lower middle-income countries. However, there is a lack of guidance and standards for monitoring and evaluation (M&E) of EPHS implementation. This paper is the final in a series of papers reviewing experiences using evidence from the Disease Control Priorities, third edition publications in EPHS reforms in seven countries. We assess current approaches to EPHS M&E, including case studies of M&E approaches in Ethiopia and Pakistan. We propose a step-by-step process for developing a national EPHS M&E framework. Such a framework would start with a theory of change that links to the specific health system reforms the EPHS is trying to accomplish, including explicit statements about the ‘what’ and ‘for whom’ of M&E efforts. Monitoring frameworks need to consider the additional demands that could be placed on weak and already overstretched data systems, and they must ensure that processes are put in place to act quickly on emergent implementation challenges. Evaluation frameworks could learn from the field of implementation science; for example, by adapting the Reach, Effectiveness, Adoption, Implementation and Maintenance framework to policy implementation. While each country will need to develop its own locally relevant M&E indicators, we encourage all countries to include a set of core indicators that are aligned with the Sustainable Development Goal 3 targets and indicators. Our paper concludes with a call to reprioritise M&E more generally and to use the EPHS process as an opportunity for strengthening national health information systems. We call for an international learning network on EPHS M&E to generate new evidence and exchange best practices.

**Summary box**

- Monitoring and evaluation (M&E) plans for essential packages of health services (EPHS) implementation should not be an afterthought—they should be integrated into the universal health coverage (UHC) policy process from the very beginning.
- The EPHS M&E process, while focused narrowly on implementation of the EPHS itself, should be aligned with the global monitoring framework for UHC and the overall national health information system structures and processes building from the Sustainable Development Goal (SDG) 3.8.1 and 3.8.2 indicators on service coverage and catastrophic expenditures, respectively.
- Because of challenges in identifying the causal effects of complex reforms on distal health outcomes, evaluation activities should be focused on changes in service coverage and financing of high-priority health services that can serve as ‘tracers’.
- While there is international consensus on indicators for UHC systems, there is no such consensus on indicators for EPHS implementation.
- To address this gap, we propose that countries use a combination of ‘core’ indicators, drawing on SDG 3.8.1 and 3.8.2, and dynamic, country-specific indicators that reflect the current EPHS implementation strategy and local needs.
- Measurement should not reduplicate other data collection efforts; it should generally be integrated into routine activities with existing staff, especially during the monitoring phase, and it should be driven and owned by the national ministry of health rather than development partners or consultants.

## Introduction

 Essential packages of health services (EPHS) have risen to prominence in low-income and middle-income countries (LMICs) as a means of delivering on Sustainable Development Goal 3.8 and national commitments to achieve universal health coverage (UHC).^1 2^ A major threat to their usefulness is that development and implementation processes have historically paid little attention to monitoring and evaluation (M&E) efforts.^3^ Consequently, there is a lack of empirical, country-derived precedent on how to conceptualise and execute M&E activities specific to EPHS-related reforms. Resource-limited countries face unique challenges in tracking the implementation and impact of their EPHS, while the proliferation of stakeholders with different M&E requirements, for example, external donors, national ministries of health, district health administrative offices and international normative bodies, limits the transferability of lessons from high-resource settings.^4^

This paper emerged from a series of meetings on capturing lessons learnt from country-level efforts to translate the model EPHS recommended in Disease Control Priorities, third edition (DCP3). Drawing on the experience of DCP3 projects in Ethiopia and Pakistan, we summarise the state of the evidence on M&E for EPHS. Ethiopia and Pakistan were chosen from among the seven case study countries in attendance (others being Afghanistan, Somalia, Kenya, Zanzibar, and Sudan) because they were the farthest along in development of their EPHS M&E frameworks. We then propose a generic framework for EPHS M&E, including reflections on key indicator features. This framework is intended as a starting point for developing local frameworks, and it will need to be reviewed and updated as experience with EPHS M&E accumulates in the coming years. We also identify high-priority areas for future research and collective action in this area. Our intention is to stimulate new dialogue and lay out a learning agenda for practitioners, project sponsors, researchers and policymakers.

### Why a new approach?

The individual interventions and services within an EPHS exist within the larger health ecosystem, and monitoring and evaluation of these health services comes in many varieties. Interventions addressing high-burden communicable conditions are captured by disease-specific M&E efforts, frequently within the context of donor-funded initiatives. Other basic services, such as obstetric care, are tracked by routine health management information systems (HMIS). Indeed, for a very low-resource country with a limited set of interventions in its EPHS, the combination of these activities may allow for monitoring of all the included services, although in a fragmented, uncoordinated way. At the policy level, national and condition-specific strategy revision processes often include retrospective analyses of health targets, implicitly or explicitly tied to services in an EPHS. These provide countries with opportunities to take stock and inform changes to the next iteration of strategic plans. Separately, there are one-off or periodic evaluations of major system areas such as health sector performance assessments that provide additional insights.

These myriad efforts are invaluable but are insufficient to capture the implementation and impact of EPHS in the context of UHC in LMICs. An EPHS is a specific policy tool intended to motivate the rationalisation of resource allocation and change the composition of services delivered. In the context of UHC, it is also a tool to advance progressive universalism by expanding the types of health conditions for which care is available. A growing number of EPHS in LMICs are including interventions for high-burden non-communicable diseases, like cardiovascular disease and cancer, as well as acute but complex issues like emergency and surgical care. To understand whether EPHS as currently designed are an effective policy mechanism for service delivery reforms, new approaches for M&E are needed. These approaches will need to draw on existing theory while integrating classical targets of evaluation, such as commodities and measures of health status, along with measures of policy implementation. The latter is especially important in determining whether the EPHS is effectively influencing activities throughout different departments of the ministry of health, rather than simply sitting on a shelf in the planning department. The goal of this new approach is not to duplicate the immense M&E efforts already underway, but to interrogate the data collected within them in a way that allows for determining whether the resource-intensive processes involved in health benefits package revision are producing the desired impact on resource allocation, equity and ultimately the scope care that is available at little to no cost to patients. In the sections that follow, we briefly review relevant literature on EPHS M&E, reflect on EPHS M&E experiences in Ethiopia and Pakistan (two countries that recently underwent EPHS revision processes) and outline how other countries could develop their own frameworks.

### EPHS M&E in the UHC era

To supplement the experiences of the DCP3 country projects and place them in context, we searched PubMed, PAIS and a few grey literature sources known to contain information on EPHS M&E. We conducted the original search in January 2022, updated in January 2023, and focused on studies published after 2002. See online supplemental annex 3 for additional information regarding the methods used.

### Monitoring

High-income country analogues of EPHS are ‘benefits packages’ and medicines formularies that are primarily tools for determining provider payments and controlling drug costs.^5^ In LMICs, however, EPHS have a mandate to rationalise the entire suite of health services that are being (or could be) provided in the country. Often they are linked to national strategic planning exercises and, as such, outline a vision for health reforms that can help progressively realise UHC by expanding the range of publicly financed health services (eg, to address emerging challenges like cancer or cardiovascular disease) as available budgets for health increase.^6^ High-quality, timely monitoring is essential for accountability and management of health facilities, and findings from the literature support the need to leverage existing data collection efforts to the greatest extent possible, even if they provide an incomplete picture of EPHS adoption, implementation and impact.^7^

Current monitoring efforts in LMICs emerged from specific programmes or disease areas (eg, HIV/AIDS, family planning, vaccination campaigns) and efforts to strengthen national HMIS generally.^4^ In settings where resource constraints effectively limit EPHS to donor-financed interventions delivered in community and primary care settings, a robust HMIS could capture the alignment of service delivery outputs with EPHS priorities. HMIS alone, however, cannot monitor whether an EPHS as a policy mechanism is being implemented as intended (eg, EPHS dissemination, changes in financial flows following EPHS revisions). There is a gap in monitoring guidance for complex, integrative policy efforts such as those related to UHC, although emerging work from the field of policy implementation science offers promise.^8^ Compounding this challenge is the fragmentation of financing and service provision mechanisms. For example, in the most recent resource-mapping exercise in Malawi, 185 sources of funding were identified, which flowed through 226 implementing agents.^9^ Existing approaches to routine monitoring that are tied to specific development projects and global health initiatives may not be meaningful for EPHS M&E.

### Evaluation

We found seven publications evaluating EPHS in LMICs.^10^,^16^ Six papers compared the contents of an EPHS with either a normative set of recommended services^12 14 15^ or assessed the extent to which the EPHS development process reflected an overarching set of aims (eg, human rights).^11 13 16^ One study assessed a set of service delivery indicators to understand the impact of EPHS on clinical or health outcomes.^10^ Beyond systematic evaluations, information on EPHS effectiveness surfaced in case studies and programme reports.^17 18^ The publications on EPHS implementation discussed post-policy adoption, one-time evaluation activities that use a range of methods. We did not find any instances of formal impact evaluations being integrated into EPHS planning and design, but to the extent that these occur they are likely to be captured within national policy processes and thus would not have been picked up by our search method.

## Country experiences

Boxes1  2 summarise the experiences with EPHS M&E in two DCP3 country projects, illustrating different potential approaches. Ethiopia took a parsimonious approach to M&E that relies heavily on population surveys^19 20^ and trends in health outcomes reported by the Global Burden of Disease study. Other countries with resource-limited information systems might choose such an approach, which is feasible in nearly all contexts and requires limited set-up or additional EPHS-specific M&E investment. Still, survey coverage is imperfect, and it is difficult to reliably correlate changes in deaths and disability-adjusted life-years to specific EPHS measures, making this approach suboptimal.

Box 1 **Essential packages of health services (EPHS) monitoring and evaluation (M&E) approach in Ethiopia**

The Federal Ministry of Health of Ethiopia underwent an EPHS revision process that was completed in 2019. This process involved 35 consultative workshops with numerous stakeholders and resulted in a list of about 1000 interventions to be included in the EPHS, with just over half of these deemed high-priority and thus free of charge. (Unlike in Afghanistan and Pakistan, the Ethiopia process drew on a range of sources for candidate interventions, beyond the Disease Control Priorities, third edition model lists.) Eregata *et al* summarise the deliberative process and outcomes.^19^

M&E plans were developed later in the EPHS reform process, and M&E for the EPHS is nested within a larger M&E framework for all of Ethiopia’s Health Sector Strategic Plan goals.^20^ Ethiopia’s framework relies heavily on population-based surveys supplemented by other data sources like health information systems and National Health Accounts data (online supplemental annex 1).

To track the universal health coverage-related objectives of the essential packages of health services, the Ministry chose 16 tracer service coverage indicators that were aligned with the WHO Service Coverage Index and Sustainable Development Goal 3.8.1. Financial risk protection indicators are also included (see online supplemental annex 1). The overarching M&E framework also includes a proposed list of tracer indicators to explicitly monitor equity of service provision across several dimensions (including sex, wealth, geography) during the EPHS implementation timeframe.^20^

The Ministry intends to evaluate the impact of the EPHS by tracking annual estimates of age-standardised death and disability-adjusted life-year rates using estimates from the Global Burden of Disease study, with 2019 as the baseline year for evaluation (online supplemental annex 1). The framework also includes mechanisms for assessing how the EPHS has been adopted within various strategy and planning activities, such as the national essential drugs list and development/revision of clinical guidelines.

Box 2 **Essential packages of health services (EPHS) monitoring and evaluation (M&E) approach in Pakistan**

Over 2017–2018, Pakistan’s Ministry of National Health Services, Regulations & Coordination led a 2-year process to develop a national-level EPHS. Because of the federal and decentralised design of the health system, this EPHS is intended as a model for contextualised, provincial-level EPHS. The latter process is currently underway, with early stage implementation in selected districts. As in Ethiopia, the national M&E framework is intended to align with Pakistan’s global commitments, principally to Sustainable Development Goal (SDG) 3.8.1 (service coverage index) and SDG 3.8.2 (financial protection).

The EPHS M&E framework development process was a detailed consultative process that included provincial governments, development partners (including UN agencies) and international academic institutes such as London School of Hygiene and Tropical Medicine. The M&E framework is organised around a results chain model that includes the six components of the WHO health systems framework. The cardinal principles used in developing the framework included:

1. The district as the primary ‘unit’ of implementation and of M&E.
2. Enhanced use of district-level routine data (ie, existing health and management information systems) for monitoring, complemented by provincial-level and national-level data.
3. A careful approach to selection and use of monitoring indicators, ensuring they can all be collected and reviewed regularly (online supplemental annex 2).
4. Monthly, quarterly and yearly benchmarks for EPHS monitoring.
5. Use of rapid and targeted special data collection activities in the yearly monitoring activities; examples include short client exit surveys, community catchment surveys across the served populations of primary healthcare facilities and other data to assess ‘effective coverage’.
6. Taking a system-wide approach for monitoring rather than focusing on the EPHS; the rationale for this approach was to integrate universal health coverage-related efforts into the existing health system, including its M&E function.

These principles apply particularly to monitoring. A detailed evaluation is planned after 3 years of implementation and will involve additional survey data collection (eg, facility surveys, client exit surveys and qualitative assessments of process indicators).

Pakistan is pursuing a more ambitious approach.^14^ M&E efforts will use a broader array of domestically generated, service delivery-focused indicator data collected via existing, strengthened national and subnational health information systems. These monitoring data will be aggregated up to evaluative metrics. Other countries inclined towards this approach would need to ensure sufficient resources for developing and maintaining such a system. The increased costs are balanced by the potential benefits of (i) leveraging the EPHS process to strengthen much-needed existing health information system infrastructure at the local and national levels and (ii) generating data that provide a compelling case for the benefits of the EPHS on equity, financial risk protection, societal trust and health outcomes.

## Key issues and unknowns in EPHS M&E processes

Logic models, used in both Pakistan and Ethiopia’s EPHS monitoring plans, and theories of change commonly used in programme monitoring more broadly^21^,^23^ provide a starting point for EPHS monitoring but suffer from two limitations. On the one hand, the simplified, linear approaches used to track single disease initiatives are inadequate for effectively monitoring the complex, systems-level objectives of health benefits packages. On the other hand, the existing EPHS-specific guidance from better-resourced countries requires extensive, highly detailed data that are often unavailable in lesser-resourced countries.

When it comes to evaluation, available empirical data are even more scarce. The cross-sectoral, decentralised nature of an EPHS makes an integrated evaluation unlikely since it is not any one department’s domain; indeed, the lack of existing literature and empirical examples underscores this point. However, unlike monitoring, existing evaluation and implementation models and tools could be readily adapted to an EPHS.

Application of current M&E frameworks to an EPHS runs the risk of going straight from policy formulation to measuring changes in service coverage and health outcomes, skipping the intermediary processes essential for understanding and improving the implementation of EPHS. We need a better understanding of the mechanisms of EPHS operation on health systems and the determinants of EPHS implementation, as distinct from broader health system and socioeconomic factors.

## Proposed approach to EPHS M&E

Given the lack of a systematic approach to monitoring and evaluating the implementation of EPHS, we propose the following stepwise approach.

### Step 1: develop theories of change

**Figure 1:**
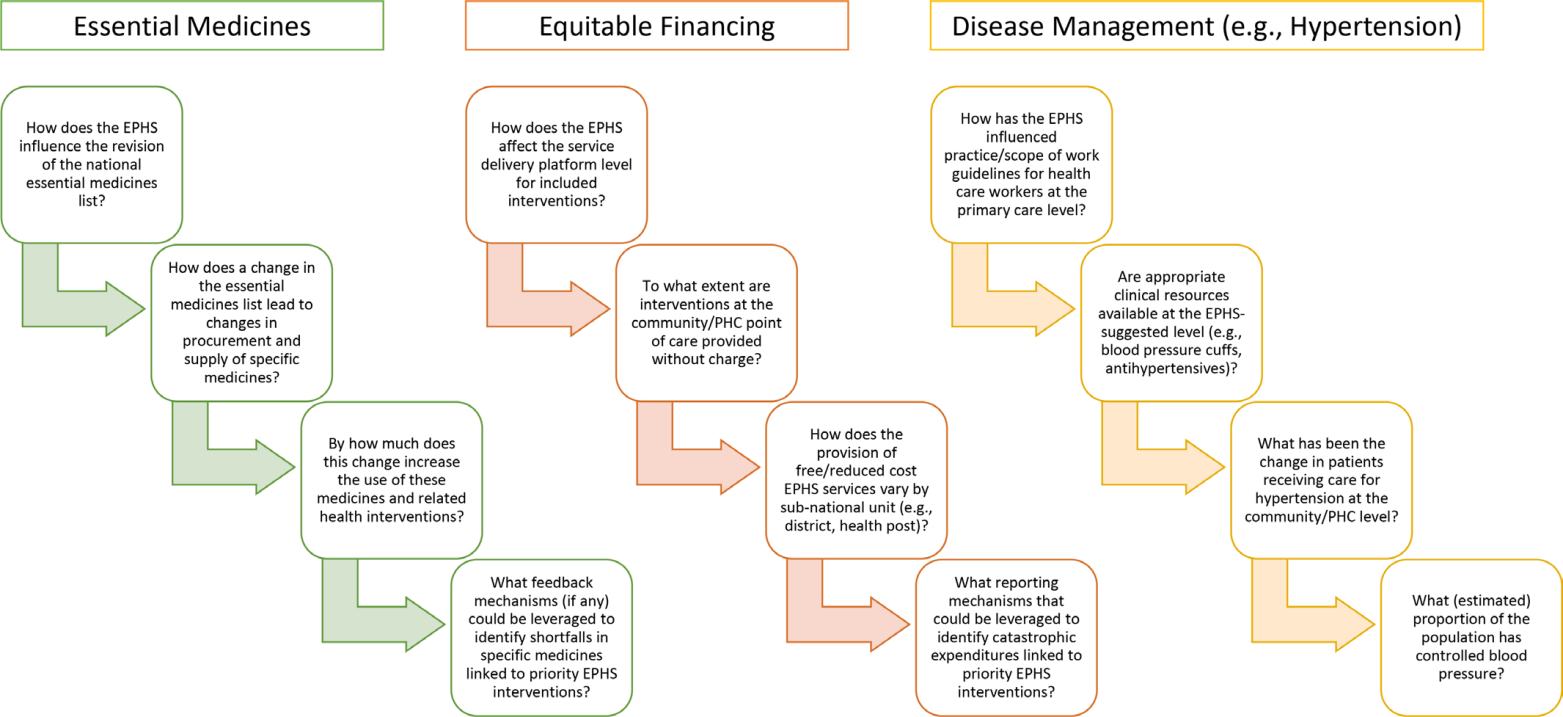
Sample questions or tracer indicators based on theories of change for essential packages of health services (EPHS) reform. PHC, primary health care.

Figure 1 presents examples of simple theories of change that illustrate how EPHS-related reforms might influence health and systems processes and outcomes that ought to be captured through M&E activities. It shows the types of questions a detailed theory of change might ask relating to priority areas for UHC and the EPHS, such as essential medicines, equitable financing and management of chronic diseases like hypertension. Local theories of change will need to include the roles and obligations of non-government actors where applicable. For example, external partners may be providing financial support for EPHS interventions, or private-sector physician groups might have a contractual arrangement with the government to provide certain essential services. *Overall, the objective of the theory-of-change exercise is to help the ministry of health determine ‘what’ is being monitored or evaluated, and for whom (ie, government or otherwise) M&E activities are being undertaken*.

### Step 2: create an EPHS M&E framework

The most common approach to M&E framework development is a results chain or logic model, which links programme inputs, activities, outputs, outcomes and impact(s) in a linear and causal manner.^21 24^ This approach is ideal for standalone health programmes of low-to-moderate complexity.^25 26^ However, it is unclear how applicable this linear, unidirectional approach is for whole-system reforms such as those implied in EPHS exercises. A retrospective look at the Pakistan experience in coming years could provide insight into the usefulness of this approach (see box 2).

The framework for EPHS monitoring and the framework for EPHS evaluation should be distinct and separate documents, although they should be aligned with each other as well as with the overall health sector M&E strategy. Monitoring is understood as an ongoing effort that uses routine data and existing staff to ensure the EPHS reforms are being implemented and corrected as needed to achieve the intended objectives. *Hence, the EPHS monitoring framework should place relatively greater emphasis on policy processes and intermediate indicators so that emergent implementation shortfalls can be quickly identified and remediated*. By contrast, evaluation is understood as a periodic effort usually covering a period of at least 12 months that builds onto monitoring activities with specialised and one-off data collection to ensure that the planned reforms are achieving their intended outcomes. *Hence, the EPHS evaluation framework should place relatively greater emphasis on measures of service coverage, health status, client satisfaction and so on, to build a coherent understanding of the effectiveness of the EPHS reforms* and their effect on populations and health system performance.

#### Monitoring

Monitoring frameworks should start from the recognition that much work has been done to strengthen health information systems in LMICs and that many countries are regularly reviewing and revising their national and subnational indicators.^27^,^29^ EPHS monitoring needs to determine how these existing data collection efforts, along with expensive ongoing surveys and health indicator databases, can be combined to understand EPHS implementation, rather than create novel indicators (see below).^7^ Developing recommendations on procedures that could be used to identify tracer indicators, for example, would be beneficial.

Monitoring of the EPHS should be done in multiple dimensions (see ‘Step 1: develop theories of change’ section). Ministries need to monitor the content of the package itself and the process used for its development. Relevant characteristics to consider include responsiveness to local needs, inclusiveness, the extent to which the delivery and organisation of services match the implementation arrangements with the health system and feasibility.^30^ Without capturing these metrics over time, it is difficult to determine whether implementation failures are due to a lack of acceptability or adoption of the EPHS (eg, among subnational planning teams), limited demand for mismatched services, insufficient resources or other factors.

The EPHS is fundamentally an evidence-informed tool to advance the UHC agenda, primarily via client interactions with the healthcare system. Prior assessments of EPHS^12 13^ have been done retrospectively, focusing on the nationally defined package. These assessments did not consider adaptations made, formally or informally, at subnational levels of service planning, or the EPHS’s impact on utilisation or out-of-pocket (OOP) costs. EPHS development is an ongoing, adaptive process, and national and subnational health contexts are expected to change over the EPHS life course (5–10 years), altering the assessment of feasibility, costs and so on. Ongoing monitoring of policy adaptation is critical.

The Pakistan experience shows the importance of tracking quality of care as an early bellwether for monitoring client experience and health system responsiveness. Traditional quantitative monitoring approaches may not be fit for this purpose, and more theoretical work is needed to understand how to integrate qualitative methods into routine monitoring efforts.

#### Evaluation

Insights from the field of implementation science can help fill the gap in linking the EPHS to changes in resource allocation, service delivery and ultimately health outcomes. For example, the Reach, Effectiveness, Adoption, Implementation and Maintenance (RE-AIM) framework has been widely used in many country-specific and disease-specific applications. RE-AIM seeks to identify, and where possible quantify, the ‘active ingredients’ of a programme that translate directly into favourable outcomes of UHC for the populations served.^31^ RE-AIM could be applied to the implementation of the EPHS in general (ie, understanding how it is being used by district managers) or to a series of specific tracer interventions (such as safe delivery) that are linked to the selected indicators (table 1).

**Table 1:** Use of RE-AIM for EPHS evaluation in general and for evaluation of the intervention ‘safe delivery’ (as a maternal health services tracer) in particular

Construct	Application to EPHS in general	Application to specific service, safe delivery
Reach	% of population covered* by facilities that use EPHS	% of population in need* receiving safe delivery services
Effectiveness	Change in service delivery (qual†)+out-of-pocket costs (quant)	Change in mortality and out-of-pocket costs for facility delivery (quant)
Adoption	% of units‡ adopting EPHS	% of units‡ adopting safe delivery service
Implementation	Level of fidelity to EPHS overall (eg, % of services provided)	Level of fidelity (quality) of core components of safe delivery
Maintenance	Sustainment of adoption/implementation over time	Sustainment of adoption/implementation over time

* Calculation of coverage would be a population-weighted average based on utilisation data and measures of adoption.

† Since an EPHS reform might continue some interventions from a previous EPHS and add or remove others, ‘effectiveness’ would need to be a holistic, qualitative assessment of how effective the EPHS reform was in *actually* changing clinical practice.

‡ ‘Units’ can refer to districts, facilities or individual providers depending on the needs of the particular application.

EPHS, essential packages of health services; qual, qualitative; quant, quantitative; RE-AIM, Reach, Effectiveness, Adoption, Implementation and Maintenance.

While RE-AIM does not usually include a specific assessment of equity impact, such an assessment could flow naturally out of the application of RE-AIM across different subnational units. Specifically, population levels of each of the (quantitative) RE-AIM indicators could be disaggregated by province/state or a comparable measure of socioeconomic/demographic status, such as through a geospatial analysis of HMIS or DHS data. In principle, results could also be stratified by gender, income or other dimensions (depending on the service area), although such stratifications would probably require additional client-level data collection via population surveys (eg, benefit incidence analysis),^32^ which could prove costly and labour-intensive in some circumstances. Establishing or expanding health records systems that can cover the entire population could also provide additional insights into the equity impact of the EPHS, although at a considerable cost.

### Step 3: select indicators

The modern concept of an EPHS is that of a policy instrument that helps achieve the SDGs, including the target relating to UHC (3.8). All countries are expected to report on two indicators related to the UHC: SDG 3.8.1 (service coverage index) and SDG 3.8.2 (financial protection) (box 3).^33 34^

Box 3 **Sustainable Development Goal 3 universal health coverage indicators**

**3.8.1** Coverage of essential health services (defined as the average coverage of essential services based on tracer interventions that include reproductive, maternal, newborn and child health, infectious diseases, non-communicable diseases and service capacity and access, among the general and the most disadvantaged population).

**3.8.2** Proportion of population with large household expenditures on health as a share of total household expenditure or income.

Pakistan and Ethiopia have integrated these two measures into their M&E frameworks, but many countries are not currently tracking even these most basic indicators, so efforts to improve national-level EPHS monitoring and reporting must start here.

We propose that M&E of EPHS implementation should use two sets of indicators. The first set, or ‘core’ indicators, would be based on the SDG 3.8 indicators, including the indicators used to compute the WHO’s Service Coverage Index (SCI), and used in nearly all countries unless there are compelling epidemiological reasons otherwise. The second set, or ‘dynamic’ indicators, would be based on the local context and specific to the reforms that the EPHS is trying to achieve. For example, breast cancer is not included in the SCI, but it is increasingly a priority for many countries. A country that introduces or significantly expands a breast cancer programme as part of an EPHS process might then include a dynamic indicator related to breast cancer screening or treatment access. There are several sources of available UHC indicators that have been used in research and implementation in LMICs that could serve as a starting point.^35 36^

Regardless of the sources of core and dynamic indicators, they should leverage ongoing data collection activities whenever possible, and the M&E needs of the EPHS should be seen as an opportunity to improve routine data collection systems. The challenges countries have experienced in reporting on the SDG indicators are a telling indicator of the depth of the need for greater investment in human, technological and financial resources.^37^ Furthermore, to minimise the risk of adding to already high data collection burdens, we recommend focusing on a limited number of tracer conditions and non-service-delivery components (eg, supply chain strengthening, financing system, etc) and their associated signal indicators. The choice of dynamic indicators should also be linked to the theory of change created during the EPHS development process (see ‘Step 1: develop theories of change’ section).

In the context of UHC and the EPHS, M&E of financial protection outcomes is particularly important. Measures of financial protection need to be aligned with the reality of fragmented, non-fungible health resource flows in many countries. For example, most catastrophic health expenditure worldwide is from non-communicable diseases,^38^ but many of the most expensive (and highest financial risk) interventions may not be included in the EPHS, especially in low-income countries. Efforts need to be made to estimate OOP spending on interventions included in the EPHS rather than OOP spending in general, since the latter may not capture the intended effect of the EPHS, that is, to reduce OOP spending on interventions in the package.

Finally, we draw a distinction between the set of measures necessary for routine tracking of the provision of comprehensive, high-quality healthcare to all citizens—the M&E function of the health system in general—with the much smaller subset of indicators required to monitor the implementation and effectiveness of an EPHS as a policy tool. As a complement to aggregate, quantitative indicators, countries should also institutionalise data collection activities that capture policy processes and the rollout of new services (ie, early policy implementation). For example, key informant interviews conducted among EPHS implementers to better understand how the EPHS is being used (or not) and what determinants of non-use are amenable to intervention.

The framework described here is intended to be a first, not a final, offering on how to extend M&E theory to understand EPHS impact. Additional theoretical work to integrate equity considerations more fully at each step is needed. The theory and its components will also need to be validated through empirical work in countries revising their EPHS.

## A call to action

Most guidance on M&E in LMICs is either aimed at strengthening national health data collection systems or follows from standalone health programmes that address specific topics like HIV/AIDS^24^ or child health.^39^ Little has been published to date on M&E specific to understanding the impact of EPHS. Current M&E tools are inadequate for providing practical, actionable direction on how to evaluate the design and implementation of EPHS as policy instruments and monitor their ongoing rollout in an affordable, timely way. The resulting risk is that departments tasked with EPHS M&E will default to broad health data collection efforts with limited adaptation or integration into an EPHS-specific theory of change and M&E framework.

In this paper, we lay out an approach to developing EPHS M&E frameworks intended to keep these needs front and centre. Ideally, political buy-in for EPHS-related reforms could provide an opportunity to mobilise additional government resources to build out routine information systems, both supporting EPHS implementation and benefiting the M&E function more broadly. Strong HMIS can be leveraged for evaluations, so efforts to build HMIS capacity should be coordinated with EPHS-specific process and implementation data needs.^40^ Additionally, considering the paucity of evidence on the relationship between EPHS and improved health outcomes in LMICs, global health research funders should consider supporting a limited number of high-quality external evaluations of EPHS.

Our approach has several practical and theoretical limitations. A full systematic search of the literature on EPHS M&E was beyond our remit and is a weakness of our findings presented here. Considering the scarcity of publications in the peer-reviewed literature, a comprehensive review that focuses particularly on grey literature and policy documentation would be immensely valuable and fill an important gap in understanding the different tools that are being applied to EPHS policy implementation M&E in practice. We are further limited by our focus on seven DCP3 country projects. Future efforts integrating lessons from non-DCP3 countries, particularly those with longer EPHS histories like Malawi and Thailand, would provide valuable insight into effective strategies for EPHS implementation M&E. Our proposed way forward underscores the need for a ‘learning agenda’ built around the experiences of countries undertaking EPHS reforms. International organisations and philanthropies committed to supporting national EPHS development should strongly consider investing in an international learning network that could help to harmonise methods, tools and reporting on country projects and help identify and disseminate best practices.

## Supplementary material

10.1136/bmjgh-2022-010726 online supplemental file 1

10.1136/bmjgh-2022-010726 online supplemental file 2

10.1136/bmjgh-2022-010726 online supplemental file 3

## Data Availability

No data are available.
