# Supplementary material for: Monitoring and evaluating the implementation of essential packages of health services
Source: BMJ Glob Health. 2023 Mar 28;8(Suppl 1):e010726. doi: 10.1136/bmjgh-2022-010726 (PMC10069525; doi:10.1136/bmjgh-2022-010726)
Supplement: online supplemental file 1 [file bmjgh-2022-010726supp001.pdf]

Annex 1. Components of Ethiopia’s M&E framework

Table 1a: Improvement/change in quality UHC service coverage

NO	*Major group*	*Tracer indicator*	*Description*	*Data source*
1	*RMNCH*	Family planning	Demand satisfied with modern method among women 15- 49 who are married or in a union	EDHS
2	*RMNCH*	Pregnancy care	Average coverage of 4 or more antenatal visits and skilled birth attendance	EDHS
3	*RMNCH*	Full child immunization	One-year-old children who have received 3 doses DPT containing vaccine	EDHS
4	*RMNCH*	Child treatment	Care seeking behavior for children with suspected pneumonia	EDHS
5	*Infectious diseases*	TB treatment	TB cases detected and cured	WHO
6	*Infectious diseases*	HIV treatment	People living with HIV receiving ART	HMIS
7	*Infectious diseases*	Malaria prevention	Population at risk sleeping under insecticide treated bed nets	MIC
8	*Infectious diseases*	Improved water and sanitation	Average coverage of households with access to improved water and sanitation	EDHS
9	NCDs	Treatment of CVD	Prevalence of raised blood pressure	STEPs
10	NCDs	Management of DM	Prevalence of raised blood glucose	STEPs
11	NCDs	Cervical cancer screening	Cervical cancer screening among women 30-49	STEPs
12	NCDs	Tobacco control	Adults age ≥ 15 years not smoking tobacco in last 30 days	STEPs
13	Service capacity and access	Hospital access	In-patient admissions per capita	HMIS
14	Service capacity and access	Health worker density	Health professionals per capita physicians, psychiatrists, and surgeons	HMIS
15	Service capacity and access	Access to essential medicines	Average proportion of WHO-recommended core list of essential medicines present in health facilities	SPA
16	Service capacity and access	Health security	International Health Regulations core capacity index	FMOH

Table 1b. Financial risk protection core indicators

NO	Tracer indicator	Description	Data source
1	Proportion catastrophic out-of-pocket expenditure	Proportion of households with catastrophic out-of-pocket health expenditure exceeding 40% of non-food total expenditure	NHA
2	Incidence of medical impoverishment		NHA
3	Proportion of households enrolled in community based health insurance (CBHI)	Number of households in the district enrolled in the CBHI scheme in the year divided by the total number of households in the district	NHA

Table 1c. Improvement /change in burden of disease as measured by Age-standardized death rate and DALYs

Rank	*Causes of death or injury (GBD Level - 2)*	*2019* Deaths	DALYs	*2020* Deaths	DALYs	*202x* Deaths	DALYs
1	Maternal and neonatal disorders						
2	Respiratory infections and tuberculosis						
3	Enteric infections						
4	Other infectious diseases						
5	Other non-communicable diseases						
6	Nutritional deficiencies						
7	HIV/AIDS and sexually transmitted infections						
8	Cardiovascular diseases						
9	Neoplasms						
10	Mental disorders						
11	Unintentional injuries						
12	Digestive diseases						
13	Neurological disorders						
14	Self-harm and interpersonal violence						
15	Musculoskeletal disorders						
16	Diabetes and kidney diseases						
