# Supplementary material for: Monitoring and evaluating the implementation of essential packages of health services
Source: BMJ Glob Health. 2023 Mar 28;8(Suppl 1):e010726. doi: 10.1136/bmjgh-2022-010726 (PMC10069525; doi:10.1136/bmjgh-2022-010726)
Supplement: online supplemental file 2 [file bmjgh-2022-010726supp002.pdf]

Annex 2. Indicators used in Pakistan's M&E framework

Summary of Key Indicators (monthly and quarterly)					
**S. No.**	**Sub-domain**	**BHU**	**RHC**	**CHC**	**MCH Centers**
1	Filled Post Index				
2	Essential Medicines/vaccine availability Index				
3	Essential Equipment Availability and Functionality Index				
5	HR Availability (% Filled)				
	Management Staff				
	Medical Staff				
	Paramedics				
	Support Staff				
6	Availability of services (hours of provision) for Basic/C- EmONC services Index				
7	Monthly report submission on regular basis (LHWs)				
8	Delivery of supplies regularly and in required quantities (LHWs) through tracer items list				
9	PHC services utilization rate (Maternal Health)				
10	PHC services utilization rate (Child Health)				

Definitions

HR Filled Index: It is a composite index of sanctioned posts filled against sanctioned posts as per the UHC-BP and according to the type of facility.

Essential Medicines/vaccine availability Index: It is a composite index of essential medicines and vaccines availability as per the UHC BP. A set of 10-20 essential medicines (depending upon the type of facility) and mandated EPI vaccines against 10 diseases (from the DoH essential drug and vaccines list) are to be used to assess the availability at PHC facility level.

Essential Equipment Availability Index: It is a composite index of essential equipment availability as per the UHC-BP at BHU, CHC, RHC and MCH centers. A total of 10-20 selected equipment items (depending upon the type of facility) are to be used to ascertain the availability of equipment.

	**STAFF**	**BHU**			**RHC**			**CHC**			**MCH**		
		**Sanctioned**	**Vacant**	**Filled**	**Sanction**	**Vacant**	**Filled**	**Sanctioned**	**Vacant**	**Filled**	**Sanctioned**	**Vacant**	**Filled**
		**#**	**#**	**%**	**#**	**#**	**%**	**#**	**#**	**%**	**#**	**#**	**%**
1	Medical Staff												
2	Paramedics												
3	Support Staff												

Medical staff category is suggested to include the Medical Officer (in-charge), and Women Medical Officer. The para-medical staff category included LHV, Medical Technician, Dispenser, Vaccinator and CDC supervisor. Support staff included sanitary inspector, Naib Qasid, Chowkidar, Sanitary worker and computer operator(s)

Summary of Human resource that is to be collated on monthly and quarterly basis

Detailed Checklists to be filled on a monthly basis from each primary health facility

**Type of facility**	**BHU**<br>**CHC**<br>**RHC**<br>**MCH center**	**Date:** ______________________________<br>**Name of Monitor:** ____________________<br>**Name of facility:** _____________________

**Monitoring and evaluation Checklist**	**BHU**		**CHC**		**RHC**	
	**Yes**	**No**	**Yes**	**No**	**Yes**	**No**
**HEALTH EDUCATION AND PROMOTION**						
**a) HYGIENE PROMOTION, WATER AND SANITATION**						
**1. Proper toilet use and hand washing practices.**	☐	☐	☐	☐	☐	☐
**2. Advice on making water safe for drinking and storage.**	☐	☐	☐	☐	☐	☐
**b) MNCH AND FAMILY PLANNING**						
**1. Skilled birth attendants.**	☐	☐	☐	☐	☐	☐
**2. Early initiation of breastfeeding.**	☐	☐	☐	☐	☐	☐
**3. Family Planning methods.**	☐	☐	☐	☐	☐	☐
**c) CHILD HEALTH & DEVELOPMENT**						
**1. Managing diarrhoea at home.**	☐	☐	☐	☐	☐	☐
**2. Growth/development monitoring.**	☐	☐	☐	☐	☐	☐
**d) NUTRITION**						
**1. Iron & folic acid supplementation of pregnant / lactating women.**	☐	☐	☐	☐	☐	☐
**2. Weaning after 6 months of age under IYCF guidelines.**	☐	☐	☐	☐	☐	☐
**CARE PROVISION**						
**a) MATERNAL HEALTH**						
**1. ANC (Screening for high risk).**	☐	☐	☐	☐	☐	☐
**2. NATAL CARE (Normal Delivery with Forceps/MVA).**	☐	☐	☐	☐	☐	☐
**3. NATAL CARE (Ambulance services).**	☐	☐	☐	☐	☐	☐
**4. PNC (Follow-up HH visit within 40 days).**	☐	☐	☐	☐	☐	☐
**b) CHILD HEALTH**						
**1. Neonatal Examination within 72 hours.**	☐	☐	☐	☐	☐	☐
**2. EPI Vaccination services – as outreach services**	☐	☐	☐	☐	☐	☐
**c) FAMILY PLANNING**						
**1. Provision of short-term methods (Condoms, pills).**	☐	☐	☐	☐	☐	☐
**2. Provision of short-term methods (IUDs, Injectables).**	☐	☐	☐	☐	☐	☐
**d) COMMUNICABLE DISEASES**						
**1.TB notification**	☐	☐	☐	☐	☐	☐
**2.Deaths reported due to pneumonia in children under age 5**	☐	☐	☐	☐	☐	☐
**e) NON COMMMUNICABLE DISEASES**						
**1. Screening of Diabetes**	☐	☐	☐	☐	☐	☐
**2. Screening of Hypertension**	☐	☐	☐	☐	☐	☐
**f) EMERGENCY SERVICES**						
**1. First Aid**	☐	☐	☐	☐	☐	☐
**2. Patient Stabilization and referral**	☐	☐	☐	☐	☐	☐
**g) SURGICAL SERVICES**						

Type of facility	BHU<br>CHC<br>RHC<br>MCH center	Date: ____________<br>Name of Monitor: ____________<br>Name of facility: ____________

Monitoring and evaluation Checklist	BHU		CHC		RHC	
	Yes	No	Yes	No	Yes	No
1. Stitching for small wounds / injuries.	☐	☐	☐	☐	☐	☐
2. Abscess drain.	☐	☐	☐	☐	☐	☐
h) DIAGNOSTIC/LAB SERVICES						
1. TESTING I) Hemoglobin	☐	☐	☐	☐	☐	☐
II) Urine R/E	☐	☐	☐	☐	☐	☐
2. X-ray	☐	☐	☐	☐	☐	☐
i) NUTRITION SERVICES						
1. Outpatient Therapeutic Program.	☐	☐	☐	☐	☐	☐
**EMoNC SERVICERS**						
a) BASIC EmONC SERVICES						
1. Augmentation of labour by oxytocic drugs	☐	☐	☐	☐	☐	☐
2. Assisted vaginal delivery (vacuum extraction, forceps)	☐	☐	☐	☐	☐	☐
b) WHO CONDUCTS DELIVERY						
1. LHV	☐	☐	☐	☐	☐	☐
2. Medical Officer	☐	☐	☐	☐	☐	☐
c) NEWBORN CARE SERVICES						
1. Neonatal resuscitation	☐	☐	☐	☐	☐	☐
2. Clean cord care	☐	☐	☐	☐	☐	☐
**AVAILABILITY OF EQUIPMENT**						
a) GENERAL HEALTH FACILITY						
1. Computer / Printer / UPS.	☐	☐	☐	☐	☐	☐
2. Electricity backup Generator.	☐	☐	☐	☐	☐	☐
b) OPD/WMO's OFFICE						
1. Examination couch.	☐	☐	☐	☐	☐	☐
2. Weighing machine (Infant).	☐	☐	☐	☐	☐	☐
3. B.P Apparatus mercury-desk type.	☐	☐	☐	☐	☐	☐
c) DIAGNOSTICS						
1. Glucometer for blood sugar	☐	☐	☐	☐	☐	☐
2. Ultrasound	☐	☐	☐	☐	☐	☐
d) LABOUR ROOM						
1. Labour /Delivery Table with washable plastic cover.	☐	☐	☐	☐	☐	☐
2. Suction and Evacuation set (SNE).	☐	☐	☐	☐	☐	☐
3. Oxygen source (portable cylinder or central wall supply), with Mask or nasal cannula.	☐	☐	☐	☐	☐	☐
4. Examination light.	☐	☐	☐	☐	☐	☐
e) DELIVERY SET						
1. Alcohol swab.	☐	☐	☐	☐	☐	☐
2. High-level disinfected or sterile surgical gloves.	☐	☐	☐	☐	☐	☐

Type of facility	BHU<br>CHC<br>RHC<br>MCH center	Date: ____________<br>Name of Monitor: ____________<br>Name of facility: ____________					

Monitoring and evaluation Checklist	BHU		CHC		RHC	
	Yes	No	Yes	No	Yes	No
3. Artery forceps.	☐	☐	☐	☐	☐	☐
4. Episiotomy Scissors.	☐	☐	☐	☐	☐	☐
f) LADY HEALTH VISITOR'S ROOM						
1. P.V. examination light.	☐	☐	☐	☐	☐	☐
2. Fetoscope.	☐	☐	☐	☐	☐	☐
g) INFECTION CONTROL						
1. Hand washing stations.	☐	☐	☐	☐	☐	☐
2. Boiler / Autoclave.	☐	☐	☐	☐	☐	☐
h) VACCINE STORAGE						
1. Vaccine refrigerator (ILR)	☐	☐	☐	☐	☐	☐
2. Temperature log.	☐	☐	☐	☐	☐	☐
**AVAILABILITY OF SUPPLIES AND MEDICINES**						
a) SUPPLIES						
1. Disposable/ Auto-disable Syringes.	☐	☐	☐	☐	☐	☐
2. Containers for sharp disposal.	☐	☐	☐	☐	☐	☐
b) MEDICINES						
1. Inj Medroxyprogesterone acetate (DMPA).	☐	☐	☐	☐	☐	☐
2. Syp Amoxicillin (trihydrate) 125mg, 250mg/5ml.	☐	☐	☐	☐	☐	☐
3. Tab Misoprostol 200mcg.	☐	☐	☐	☐	☐	☐
4. Oral Rehydration Salt – ORS.	☐	☐	☐	☐	☐	☐
c) VACCINES						
1. Penta-valent.	☐	☐	☐	☐	☐	☐
2. Measles.	☐	☐	☐	☐	☐	☐
d) FAMILY PLANNING COMMODITIES						
1. Condoms.	☐	☐	☐	☐	☐	☐
2. Combined Oral Contraceptive (COC) Pills.	☐	☐	☐	☐	☐	☐
**ACQUISITION OF MEDICINES AND FP SUPPLIES**						
a) REASONS FOR STOCK OUTS						
1. Delayed demand submission.	☐	☐	☐	☐	☐	☐
2. Lack of storage capacity.	☐	☐	☐	☐	☐	☐
b) SOURCES OF FAMILY PLANNING COMMODITIES						
1. Department of Health/DHO office.	☐	☐	☐	☐	☐	☐
2. National/ International agencies.	☐	☐	☐	☐	☐	☐
c) SOURCE OF MEDICINES						
1. Department of Health/EDO office.	☐	☐	☐	☐	☐	☐
2. Self-procurement.	☐	☐	☐	☐	☐	☐
**FACILITY MANAGEMENT, RECORDING AND REPORTING**						
a) AVAILABILITY OF DUTY ROSTER AND DISEASE MANAGEMENT POSTERS/PROTOCOLA						

Type of facility	BHU<br>CHC<br>RHC<br>MCH center	Date: ____________<br>Name of Monitor: ____________<br>Name of facility: ____________

Monitoring and evaluation Checklist	BHU		CHC		RHC	
	Yes	No	Yes	No	Yes	No
**1. Normal Vaginal Delivery care.**	☐	☐	☐	☐	☐	☐
**2. Staff duty roster.**	☐	☐	☐	☐	☐	☐
**b) WORK COORDINATION AND SUPERVISION**						
**1. DHIS report submission.**	☐	☐	☐	☐	☐	☐
**2. Availability of supervisory visit record.**	☐	☐	☐	☐	☐	☐
**c) RECORDING AND REPORTING TOOLS**						
**1. OPD register.**	☐	☐	☐	☐	☐	☐
**2. Medicine Stock register (medicine store).**	☐	☐	☐	☐	☐	☐
**3. Family Planning register.**	☐	☐	☐	☐	☐	☐
**4. EPI register (EPI room).**	☐	☐	☐	☐	☐	☐

ANNUAL MONITORING ACTIVITIES

1. PHYSICAL INFRASTRUCTURE AND UTILITIES

S No.	Characteristic	Categories	BHU	CHC	RHC	MCH center
**1.**	**ACCESS / UTILITIES**	Metaled access road				
		Facility sign board				
		Electricity				
		Telephone				
		Functional Generator & Fuel				
**2.**	**COMPOUND**	Boundary Wall (Structure)				
		Boundary Wall (Paint/whitewash)				
		Compound gate				
**3.**	**WASTE DISPOSAL**	Rubbish pit				
		Sewerage pipe from building to external sewer/drain				
		External sewer/drain				
**4.**	**ENTRANCE TO BHU BUILDING**	Space for registration				
		Drug dispensing room				
		Waiting area for patients				
		Ramp for disabled				
**5.**	**WAITING AREA**	Covered area				
		Separate male waiting area				
		Benches at male waiting area				
		Functional ceiling fans at female waiting area				

S No.	Characteristic	Categories	BHU	CHC	RHC	MCH center
		Separate Female waiting area				
		Benches at female waiting area				
		Functional ceiling fans at male waiting area				
		Complaint/suggestion box				
6.	**TOILETS**	Toilets for staff (male)				
		Separate female staff toilets				
		Toilets for patients/attendants (male)				
		Separate female patients/attendants' toilets				
7.	**WATER SUPPLY**	Pipe with running water				
		Storage tank				
		Protected water source				
8.	**EXAMINATION ROOM**	Examination room for men				
		Examination room for women				
		Curtains/screens to ensure privacy				
9.	**LABOUR ROOM**	Well-lit				
		Ventilation				
		Attached toilet				
		Drinking water facility				
		Designated space for new-born care				
10.	**RESIDENTIAL BLOCK**	Doctors				
		Paramedical staff				
		Support staff				
11.	**GENERAL STORE**	Well-lit				
		Ventilation				
		Area for storage of sterile linen				
		Area for storage of common linen				
		Area for storage of other materials/drugs/consumables				
12.	**OTHER AREAS**	Dispensing cum store area				
		Vaccine storage and immunization area				
		BCC and family planning counsel area				
13.	**CLEANLINESS OF BUILDING**	Waiting Area				
		Consultation rooms				
		Treatment/injection rooms				

S No.	Characteristic	Categories	BHU	CHC	RHC	MCH center
		Delivery room				
		Main Pharmacy / Dispensing area				
		Toilets-patients				
		Store room				
		Delivery room				
12.	**REPAIR REQUIREMENTS OF BUILDING**	Windows and doors				
		Interior paint				
		Facility interior walls				
		Facility exterior walls				
		Floor				
		Roof condition				
		Windows and doors				
		Interior paint				

2. RANGE OF SERVICES

a. HEALTH EDUCATION AND PROMOTION

S No.	Characteristic	Categories	BHU	CHC	RHC
1.	**HYGIENE PROMOTION, WATER AND SANITATION**	Hand washing with soap			
		Proper toilet use and hand washing practices			
		Problems related to open defecation			
		Advice on making water safe for drinking and storage			
2.	**MNCH AND FAMILY PLANNING**	Importance of antenatal check-up			
		Tetanus Toxoid (TT) injections during pregnancy			
		Danger signs during pregnancy			
		Skilled birth attendance			
		Danger signs during labour			
		Consulting for post-partum examination			
		Danger signs after delivery			
		Bathing the neonate			
		Early wrapping and keeping baby warm			
		Early initiation of breastfeeding			
		Neonatal danger signs			
		Optimal period of birth spacing			
		Family Planning methods			

S No.	Characteristic	Categories	BHU	CHC	RHC
3.	**CHILD HEALTH & DEVELOPMENT**	Exclusive Breast Feeding up to 6 months			
		Continuation of Breast Feeding till 2 years of age			
		Child immunization			
		Managing diarrhoea at home			
		Growth/development monitoring			
4.	**NUTRITION**	Iron / folic acid supplementation of pregnant lactating women			
		Balanced diet for adolescents and adults			
		Weaning after 6 months of age under IYCF guidelines			
		Prevention of parasitic infections and deworming			
		Iron / folic acid supplementation of pregnant lactating women			

b. CARE PROVISION

S No.	Characteristic	Categories	BHU	CHC	RHC
1.	**MATERNAL HEALTH**	ANC (Screening for high risk)			
		ANC (TT Vaccination - Static Centre)			
		ANC (Dietary Counselling)			
		NATAL CARE (Normal Delivery with Forceps/MVA)			
		NATAL CARE (Referral for complicated delivery)			
		NATAL CARE (Ambulance services)			
		PNC (Screening for risk/complications)			
		PNC (Follow-up HH visit within 40 days)			
2.	**CHILD HEALTH**	Neonatal Examination within 72 hours			
		EPI Vaccination services – at health facility			
		EPI Vaccination services – as outreach services			
		Growth Monitoring			
3.	**FAMILY PLANNING**	Provision of short-term methods (Condoms, pills)			
		Provision of short-term methods (IUDs, Injectables)			
4.		Injury management			

S No.	Characteristic	Categories	BHU	CHC	RHC
	**EMERGENCY SERVICES**	Dog / Snake bite			
		First Aid			
		Patient Stabilization			
**5.**	**SURGICAL SERVICES**	Stitching for small wounds / injuries			
		Abscess drain			
		Circumcision			
		Back slab plaster			
		Gastric lavage			
		Catheterization			
**6.**	**DIAGNOSTIC/LAB SERVICES**	TESTING (Hemoglobin, Urine)			
		Ultrasonography			
		X-Ray			
		Other			
**7.**	**NUTRITION SERVICES**	Outpatient Therapeutic Program			
		Referral linkage with a Stabilization center at THQ/DHQ			
		Provision of nutrition supplements			

3. EMoNC SERVICES

a. BASIC EMoNC SERVICES

S No.	Characteristic	Categories	BHU	CHC	RHC
**1.**	**BASIC EmONC SERVICES**	(Parenteral) antibiotics			
		Augmentation of labour by oxytocic drugs			
		Management of Pre-eclampsia and eclampsia by sedatives			
		Manual removal of placenta			
		Removal of retained products (Manual Vacuum Aspiration (MVA), without general anaesthesia, D&C)			
		Assisted vaginal delivery (vacuum extraction, forceps)			
**2.**	**WHO CONDUCTS DELIVERY**	WMO			
		LHV			
		Midwife			
		Dai			
		MO			
		WMO			
**3.**	**NEWBORN CARE SERVICES**	Neonatal resuscitation			
		Warmth (drying, wrapping the baby and skin-to-skin contact)			
		Clean cord care			

		Early initiation of breast feeding			
		Eye care			

4. AVAILABILITY OF EQUIPMENT

S No.	Characteristic	Categories	BHU	CHC	RHC
1.	**GENERAL HEALTH FACILITY**	Ambulance			
		Electric water cooler			
		Computer / Printer / UPS			
		Electricity backup Generator			
		Fuel for running generators			
2.	**OPD/WMO's OFFICE**	Office chairs			
		Examination couch			
		Patient stool			
		Thermometer			
		Torch with batteries			
		Otoscope			
		Weighing machine (Adult)			
		Weighing machine (Infant)			
		Height measuring board			
		B.P Apparatus mercury-desk type			
		Stethoscope			
		Foetal stethoscope			
		Steam inhaler			
		Nebulizer			
		X-ray view box			
3.	**DIAGNOSTICS**	X-ray unit			
		ECG machine			
		Glucometer for blood sugar			
		Ultrasound			
4.	**LABOUR ROOM**	Labour /Delivery Table with washable plastic cover			
		Macintosh/plastic apron			
		Delivery Light			
		Normal delivery set			
		Standard surgical set (for minor procedures like episiotomy stitching)			
		Bulb Sucker			
		Fetal heart detector (Fetoscope)			
		Examination light			
		Suction and Evacuation set (SNE)			
		IUD insertion kit			
		Adult stethoscope			
		Bedpans			

S No.	Characteristic	Categories	BHU	CHC	RHC
		Blood pressure apparatus			
		Adult ambu bag and mask			
		Thermometer			
		Oxygen source (portable cylinder or central wall supply), with Mask or nasal cannula;			
		Baby weighing scale			
		Step stool			
		Suture needles			
		Partograph forms			
		Adult weighing scale			
		Manual Vacuum Aspirator (MVA)			
5.	**DELIVERY SET**	Alcohol swab			
		Blanket for wrapping the newborn			
		High-level disinfected or sterile surgical gloves			
		Episiotomy Scissors			
		Straight Scissors			
		Needle Holder			
		Artery forceps			
		Cord Clamp			
		Needle & Sutures			
		Alcohol swab			
		Blanket for wrapping the newborn			
		High-level disinfected or sterile surgical gloves			
		Episiotomy Scissors			
		Straight Scissors			
		Needle Holder			
		Artery forceps			
		Cord Clamp			
		Needle & Sutures			
6.	**LADY HEALTH VISITOR'S ROOM**	D & C instruments set			
		P.V. examination light			
		Examination couch			
		Patient stool			
		Table			
		Chairs			
		Weighing machine			
		BP apparatus			
		Stethoscope			
		Fetoscope			
		Thermometer			

S No.	Characteristic	Categories	BHU	CHC	RHC
		Torch with batteries			
		D & C instruments set			
		P.V. examination light			
		Examination couch			
		Patient stool			
7.	**INFECTION CONTROL**	Hand washing stations			
		Disinfectants			
		Boiler / Autoclave			
		Disposable syringe cutter			
		Puncture resistant container for sharps disposal			
		Bucket for soiled pads and swabs			
8.	**VACCINE STORAGE**	Vaccine refrigerator (ILR)			
		Thermometer for vaccine refrigerator			
		Temperature log			

5. AVAILABILITY OF SUPPLIES AND MEDICINES

S No.	Characteristic	Categories	BHU	CHC	RHC
1.	**SUPPLIES**	Gloves			
		Clean / Safe delivery kit			
		Disposable/ Auto-disable Syringes			
		Vaccine Syringes			
		Surgical Spirit			
		Surgical cotton			
		Gauze			
		Scalpel blades			
		Containers for sharp disposal			
		Wheel chair			
		Stretcher			
2.	**MEDICINES**	Tab Paracetamol 500mg			
		Tab Chlorpheniramine (hydrogen maleate) 4mg			
		Syp Amoxicillin (trihydrate) 125mg, 250mg/5ml			
		Syp Mebendazole 100mg/5ml			
		Tab Metformin (hydrochloride) 500mg			
		Tab Methyldopa 250mg			
		Tab Ferrous sulphate + Folic acid (60mg/400mcg)			
		Tab Misoprostol 200mcg			
		Ethynylestradiol + Norethiestradiol CO pills 35mcg +1mg			

S No.	Characteristic	Categories	BHU	CHC	RHC
		Inj Medroxyprogesterone acetate (DMPA)			
		Oral Rehydration Salt – ORS			
		Tab Cotrimoxazole (120mg or 480mg)			
		Tab Zinc 20mg/ PAC-100			
**3.**	**VACCINES**	BCG			
		OPV			
		Penta-valent			
		Measles			
		TT (Tetanus Toxoid)			
**4.**	**FAMILY PLANNING COMMODITIES**	Condoms			
		Combined Oral Contraceptive (COC) Pills			
		IUCDs			
		Injection DMPA			
		Implants			
		Condoms			
		Combined Oral Contraceptive (COC) Pills			

6. ACQUISTION OF MEDICINES AND FP SUPPLIES

S No.	Characteristic	Categories	BHU	CHC	RHC
**1.**	**REASONS FOR STOCK OUTS**	Problems of quantification			
		Delayed demand submission			
		Unavailability of buffer stock			
		Lack of storage capacity			
		Delayed supply			
		Supply less than the amount demanded			
		No procurement powers			
		Insufficient budget			
		Lack of cold-chain			
		Others: ___________			
**2.**	**SOURCES OF FAMILY PLANNING COMMODITIES**	Department of Health			
		Population Welfare Dept.			
		NP for FP & PHC(LHW Program)			
		National/ International agencies			
		Others: ____________			
**3.**	**SOURCE OF MEDICINES**	Department of Health/EDO office			
		Self-procurement			
		DSU			
		Others: ______________			

7. FACILITY MANAGEMENT, RECORDING AND REPORTING

S No.	Characteristic	Categories	BHU	CHC	RHC
1.	AVAILABILITY OF DUTY ROSTER AND DISEASE MANAGEMENT POSTERS/PROTOCOLA	Staff duty roster			
		Antenatal care			
		Normal Vaginal Delivery care			
		Postnatal care			
		Emergency obstetric care			
		Newborn resuscitation			
		Integrated Management of Newborn &Childhood Illnesses (IMNCI)			
		Family planning			
		Infection control			
		Vaccination (EPI)			
		EPI outreach plan			
		Board with list of services, opening time and emergency contacts			
		A list with all fees and possible exemptions			
		Treatment protocols for severe acute malnourished children			
2.	WORK COORDINATION AND SUPERVISION	DHIS report submission			
		Performance Review Meetings			
		Participation in District Meetings			
		District officials visit to health facility			
		Availability of supervisory visit record			
		Feedback of supervisory visits			
		Availability of quality improvement plan			
3.	RECORDING AND REPORTING TOOLS	OPD ticket			
		OPD register			
		Maternal/ Mother health register			
		Obstetric/ Birth register			
		Family Planning register			
		EPI register (EPI room)			
		Meeting register/ Facility Staff Meeting Register (Facility In-charge)			

S No.	Characteristic	Categories	BHU	CHC	RHC
		Medicine Stock register (medicine store)			
		Daily medicine expense register (OPD dispensary)			
		DHIS monthly report (Facility In-charge)			
