# Supplementary material for: Monitoring and evaluating the implementation of essential packages of health services
Source: BMJ Glob Health. 2023 Mar 28;8(Suppl 1):e010726. doi: 10.1136/bmjgh-2022-010726 (PMC10069525; doi:10.1136/bmjgh-2022-010726)
Supplement: online supplemental file 3 [file bmjgh-2022-010726supp003.pdf]

Annex 3. Literature Search Process and Results

The findings and proposed approach to monitoring and evaluation (M&E) of essential packages of health services (EPHS) are based on the lessons learnt from the experiences of ministry of health and technical advisors who developed the M&E approaches for EPHS in Disease Control Priorities 3 translation countries, described in the manuscript. To supplement the information from these practice examples we searched the peer reviewed and grey literature for published reports of EPHS-specific evaluations. The search strings and summary of results are listed below. We included results that described assessments of EPHS policy content, implementation, or impact. We excluded articles that merely described the process for designing EPHSs or were solely costing exercises of EPHSs. We conducted the original search in January 2022 and updated the search in January 2023.

<u>Peer-reviewed literature</u>
Our initial focused on Pubmed, which was selected given its health sciences focus to capture a wide selection of peer reviewed articles on monitoring and evaluating of health programs in LMICs. During the January 2023 update we added PAIS to capture journals oriented toward public policy and political sciences.

- Pubmed search string: ("health benefits package"[All Fields] OR "essential package of health services"[All Fields] OR "essential health package"[All Fields]) AND ("evaluation"[Title/Abstract] OR "monitoring"[Title/Abstract])

- PAIS search string: (“health benefits package" OR "essential package of health services" OR "essential health package") AND (noft(monitoring) OR noft(evaluation))

The Pubmed search yielded 11 results, of which 2 were included. The PAIS search yielded five results of which one was included.

We also searched Google Scholar with similar combinations of search terms adapted for the Google Scholar syntax (e.g., “health benefits package” and evaluation). We reviewed the first 30 results from each search combination and included 4 results.

<u>Grey literature</u>
We conducted a broad Google search using the term combinations below and looked at the first 20 results.

- Health benefits package + monitoring + LMIC
- Health benefits package + evaluation + LMIC
- Essential package of health services + monitoring + LMIC

- Essential package of health services + evaluation + LMIC
- Essential health package + monitoring + LMIC
- Essential health package + evaluation + LMIC

We complemented the Google search with a targeted search of the databases of three specific organizations, MEASURE Evaluation[1], the World Health Organization[2], and the National Academies of Sciences, Engineering, and Medicine[3], known to be working in the area (based on the professional experience of the author team).

References

1. MEASURE Evaluation. *Publications -- MEASURE Evaluation*. 2022 [cited 2022 March 18, 2022]; Available from: https://www.measureevaluation.org/publications.html.
2. World Health Organization. *Publications*. 2022 [cited 2022 March 18]; Available from: https://www.who.int/publications.
3. National Academies of Science, E., and Medicine. *The National Academies Press*. 2022 [cited 2022 March 18]; Available from: https://nap.nationalacademies.org/.
